# PortGISANS: nested mirror optics for a portable GISANS adapter

**DOI:** 10.1038/s41598-025-28836-3

**Published:** 2025-11-21

**Authors:** Filip Mehler, Sebastian Jaksch, Marité Cárdenas, Bert Nickel, Max Wolff

**Affiliations:** 1 Department for Physics and Astronomy, Uppsala University, 752 37 Uppsala, Sweden; 2 European Spallation Source, 221 00 Lund, Sweden; 3 Biofisika Instituta, Ikerbasque, 48940 Bilbao, Spain; 4 Faculty of Physics and CeNS, Ludwig-Maximillians-Unversität, 80539 Münich, Germany

**Keywords:** Optics and photonics, Physics

## Abstract

Grazing-incidence scattering techniques are powerful tools for studies in surface science. In particular, grazing-incidence neutron scattering (GISANS) provides unique insights in soft-matter systems and magnetic structures, in particular, if they are buried beneath a surface. However, the brilliance of neutron sources is limited hindering the method from becoming widely used. Here we present focusing nested mirror optics that improve the GISANS capabilities of SANS beamlines. Our device preserves the lateral resolution along an interface and relaxes the resolution out-of-plane, sacrificing depth-resolution. By combining ray tracing simulations with the distorted wave born approximation we show that a portable and easy to install optics increases the flux available for GISANS studies, at least, by one order of magnitude and enables straight forward experiments from free liquid surfaces.

## Introduction

The research field of surface science has grown in importance through the last decades thanks to the development of instrumentation using a range of approaches from optical to acoustic. Simultaneously, experimental surface scattering techniques have improved not only in instrumentation but also in the data analysis facilitating their use and application for non-specialized users. This has resulted in a range of studies on thin films and low dimensional systems with increasing precision and complexity^1,2^. To date, challenges remain for studying interfaces with buried structures as well as light elements and magnetism, since these topics are challenging to address with most laboratory techniques.

Neutron scattering methods combine high penetration power with the sensitivity to light elements and magnetic induction and are particularly useful for the study of thin films^3–6^. Depth profiles across surfaces and interfaces are accessible via specular neutron reflectometry (NR)^6,7^. The extracted quantity is the scattering length density (SLD), which can be directly translated into the composition of nuclei or magnetic induction. This method is well established and a multitude of fitting softwares are available. In-plane structures can be probed by off-specular and grazing incidence small angle neutron scattering (GISANS, for lecture notes we refer to Ref^8^.), depending on the length scale of interest. However, the analysis is more challenging, see e.g^9^. and established fitting tools are just emerging^10–12^. Moreover, both methods suffer from the small sample volumes probed and limited brilliance of current neutron sources. Note, this is significantly different for x-rays, where synchrotrons provide inherently narrow and well collimated x-ray beams of high intensity. As a result the method of GISAXS is more developed^1,13–16^. There are, however, some unique advantages of using neutrons over X-rays, as they allow for contrast variation by isotope substitution and their inherent spin probing magnetic samples and structures^2,5^. Particularly, for GISANS there is the possibility for studies of lateral domains in thin film materials with light elements. Examples include lipid films on substrates with controlled curvature^17^, block copolymer films^18,19^ and studies of lateral structures with magnetic properties^20–23^.

The above mentioned experimental studies have been performed either on small angle neutron scattering (SANS) instruments or neutron reflectometers. For SANS usually a point collimated beam is sent onto a sample that is tilted to grazing incidence. This results in a large beam footprint and in order not to over illuminate the sample large samples are needed to avoid a significant reduction in flux onto the sample. Reflectometers are optimised for grazing incident geometry but usually probe out-of-plane correlations using a line focus. Collimating in two directions (point focus) as required for GISANS results in reduced flux. So far, just one instrument, REFSANS at FRM-II^24^, has been built specifically for GISANS studies. On this instrument the beam is focused onto the detector rather than the sample, which again requires relatively large samples, as well as some other design constraints, which, so far, hindered a broad usage.

With improving performance of neutron mirrors beam shaping and focusing becomes more readily available. This gives more flexibility with respect to the beam size and divergence impinging onto a sample. Examples include elliptical guides^25^, and novel designs like the SELENE concept^26^. However, due to the weak interaction of neutrons with matter such guides tend to be long and bulky. For more compact devices, nested mirrors can be utilized, as shown by a recent design of elliptical and parabolic mirror stacks for neutron extraction, transport and focusing^27,28^.

The utilization of a line focused divergent beam for GISANS was recently shown on the reflectometer SuperADAM, by turning the sample by 90°, so called PI-GISANS mode^29^. This results in the line focus being along the sample normal and results in a large divergence along the direction out-of-plane but preserving the good resolution, which was then in-plane. This method was used to extract structural information about iron filled aluminium oxide pores, silica particles deposited on a wafer as well as spread out on an air/liquid interface. It was shown that good quality GISANS data could be acquired with reasonable counting times. The compromise of the method is the limited depth resolution due to the increased divergence in the out-of-plane direction. However, so far, only a few studies have attempted depth resolved GISANS measurements^2,30–32^ and it has been shown that the weak absorption of neutrons makes it very difficult to separate surface from bulk scattering^33^.

We build on the above mentioned idea and take benefit of the inherently well collimated beam on SANS instruments. We present ray-tracing simulations of a device, PortGISANS, using nested mirror optics optimized for the PI-GISANS idea. We demonstrate that the useful flux at a sample surface for GISANS measurements is increased by a factor of 12, as compared to the SANS instrument without PortGISANS. An additional benefit is that the beam gets inherently tilted downwards facilitating experiments on free liquid surfaces. Moreover, our device is very compact and easy to ship, which will allow broad usage on almost any existing SANS instrument optimizing it for GISANS. To benchmark the performance, we show simulated data on the SKADI instrument for silica nano-particles spread on a $D_2O/Si$ solid-liquid interface.

## Principle and design

**Fig. 1:**
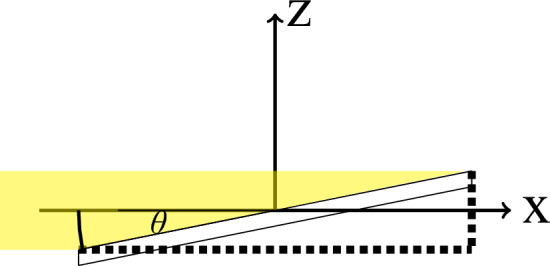
Sketch of the grazing incident beam geometry showing a narrow parallel beam impinging on a tilted sample. The small angle of incidence results in a large footprint (illumnated sample area).

On SANS instruments, a large but very well collimated beam is used together with a pinhole right in front of the sample. This beam geometry is not ideal for GISANS studies, as it results in a large footprint on a sample mounted under grazing incidence beam geometry (see Fig. 1).

The tight collimation on SANS instruments allows the use of mirrors without the need of high m-values to manipulate the beam. We use this fact to reduce the footprint and increase the flux on the sample surface by increasing the divergence along the out-of-plane direction, by optical focusing, but keeping the good collimation in the sample plane. This results in a significantly increased flux for GISANS studies, at the expense of depth sensitivity. For many applications this is a good compromise, as depth resolution is not needed, as for example in the case of single interfaces or thin films.

For a monochromatic SANS instrument, as used at reactor sources the design of such a nested optics mirror device is simple. In the case of a pulsed source and time of flight measurements, the band width of the pulse has to be considered. This is important since shorter wavelengths are harder to reflect due to the lower critical angle of total external reflection at a given interface. For the mirror assemblies described here, the cut off for the mirror at lowest incidence angle is approximately 0.5 Å, while for the mirror with highest incident angle the cut off is at 5 Å. We simulate the performance of mirrors with a m-value of 4 (for a review on supermirrors see^34,35^). Further we take spatial constrains, maximum mirror-to-sample distance of 0.5 m, and a wavelength bandwidth between 3 and 8 Å as existing on the instrument SKADI^36,37^ at the European Spallation Source (ESS). The spatial constraint limits the use of conventional single mirror optics. For example a single flat mirror inclined at $0.6 ^{\circ }$ would occupy about 1 m of space to deflect the same beam height (1 cm) as the nested design. For instruments without spatial constraints a single mirror design can be feasible and preferable to the nested approach. The instruments at ESS are designed for large band width. A nested mirror device design for SKADI is therefore more challenging than for other instruments. It can be expected that the performance on other instruments than SKADI may be even better than demonstrated here. The compact design of PortGISANS will allow easy transportation and implementation at different SANS beamlines. Further, PortGISANS inclines the neutron beam and allows GISANS experiments from free liquid surfaces in a very straight forward manner.

For a stack of flat mirrors the parameters are calculated iteratively based on the following scheme: An angle $\theta$ is set for the first mirror to hit the sample placed at 0.5 m distance and 1.5 cm below the entrance position of the top mirror

1 $$\begin{aligned} \theta (i) = \frac{arctan({\frac{z_1(i)}{X_s}})}{2} \end{aligned}$$

The end position of the mirror is set by

2 $$\begin{aligned} z_{2}(i) = z_1(i)-L*sin(\theta ) \end{aligned}$$

The next mirror is put at a height equivalent to the end of the previous mirror and an angle so the beam hits the same spot as the previous mirror.

3 $$\begin{aligned} z_{1}(i+1) = z_{2}(i) \end{aligned}$$

**Fig. 2:**
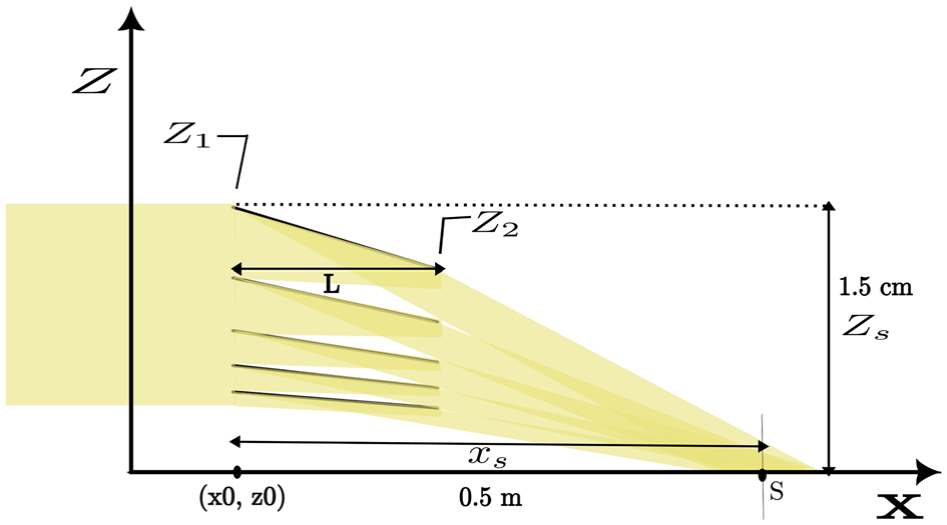
Schematics of a set of nested flat neutron mirrors diverting the beam onto a sample surface. The sample is placed at the point S, 0.5 m from the entrance point of the mirrors at $x_0$. The deflection of the beam results in a lower height of 1.5 cm of the sample below the entrance point of the top mirror.

With a mirror length of 0.2 m and 5 mirrors in the stack, a beam height of 1 cm is covered. The mirror length was chosen such that the beam size (footprint) is 10 cm at the sample position. In Fig. 2 a schematic of the flat mirror assembly including dimensions, not to scale, is shown.

Second, we assume parabolic nested mirrors of the same length of 0.2 m. The parabolic focusing results in a smaller footprint than for flat mirrors. Further shadowing effects are kept low and the divergence profile is weighted towards lower grazing incidence angles. The divergence profile at the sample depends on the height difference between the mirrors and the focus point, the distance to the focus point and the length of the mirrors. Keeping the height difference as low as possible (below the straight beam) and the distance as large as possible (limited by the space at the instrument), the angular divergence can be optimized towards lower angles. Increasing the length of the mirrors allows to divert a larger beam, but the extra amount of beam covered is mostly diverted at higher angles and increases shadowing of angles at lower grazing incidence angles. In the simulations we assume infinitely thin mirrors, which slightly overestimates the performance as for a real device the finite thickness of the mirrors will result in shadowing effects.

The top mirrors focal length is set to hit the sample at a distance of 0.5 m from the entrance of the mirror and a height difference of 1.5 cm, as assumed for the flat mirror assembly. In the case where the focal length f is much smaller than the distance from the mirrors to the focal point $x_f$ the following relation holds:

4 $$\begin{aligned} f(i) = \frac{z_{1}^2(i)}{4*x_f} \end{aligned}$$

The end point of the mirror is given by

5 $$\begin{aligned} z_{2}(i) = \sqrt{z_{1}^2(i) - L*4*f(i)} \end{aligned}$$

The next mirror is set at a height equivalent to the end of the previous mirror and with a new focal length calculated to hit the same spot as the previous mirror.

6 $$\begin{aligned} z_{1}(i+1) = z_{2} \end{aligned}$$

**Fig. 3:**
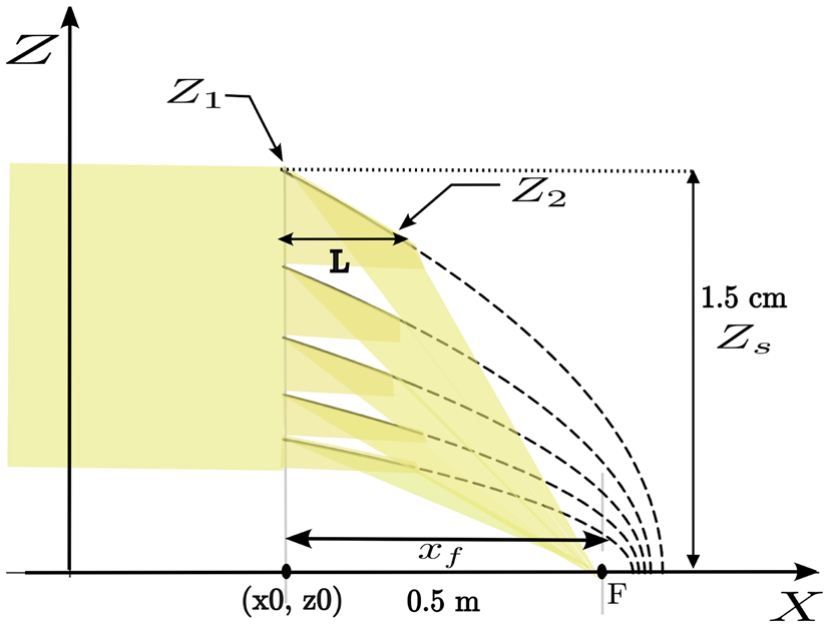
Schematics of a set of nested parabolic neutron mirrors diverting the beam onto a sample surface. The sample is placed at the point F, 0.5 m from the entrance point of the mirrors at $x_0$. The deflection of the beam results in a lower height of 1.5 cm of the sample below the entrance point of the top mirror.

Figure 3 shows a sketch of the parabolic mirror assembly. As for the flat mirrors, with a mirror length of 0.2 m and 5 mirrors in the stack, a beam height of 1.0 cm is diverted.

## Results

**Fig. 4:**
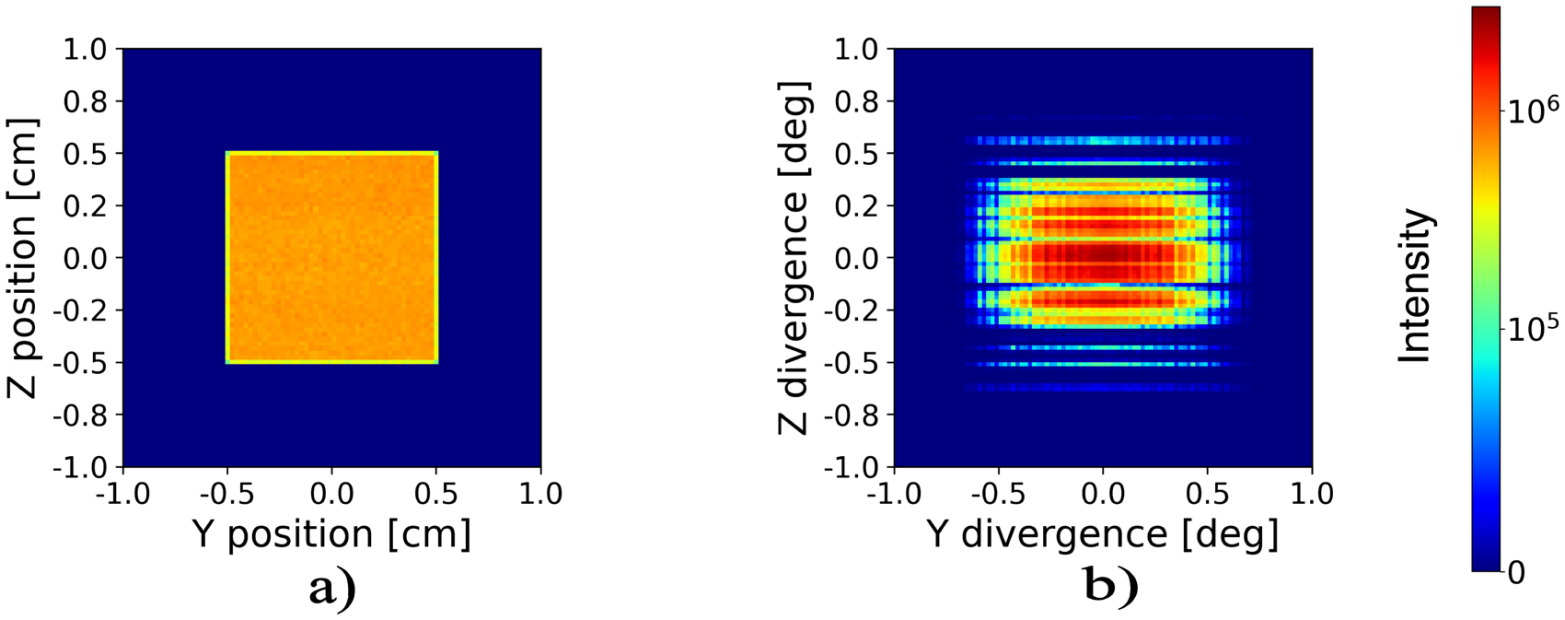
Panels (**a**) and (**b**) are the intensity and divergence distribution, respectively, at the exit of the SKADI guide used as incoming beam for the mirror assembly.

Ray tracing simulations were performed in the Mcstas package^38,39^. The simulated beam at the end of the collimation, just before the sample of the SKADI instrument^36,37^ was provided by Jaksch and represents one pulse of the ESS source at a power of 5 MW and a collimation of 4 m. The standard bandwidth between 3 and 8 Å available was used for the simulations. For further details see^37^. This beam from the SKADI model was used as input to simulate the nested mirror assemblies. In Fig. 4 panels (a) and (b) show the intensity and divergence profiles at the exit of the SKADI guide. The data confirms a very even intensity distribution across the guide as well as the narrow collimation.

**Fig. 5:**
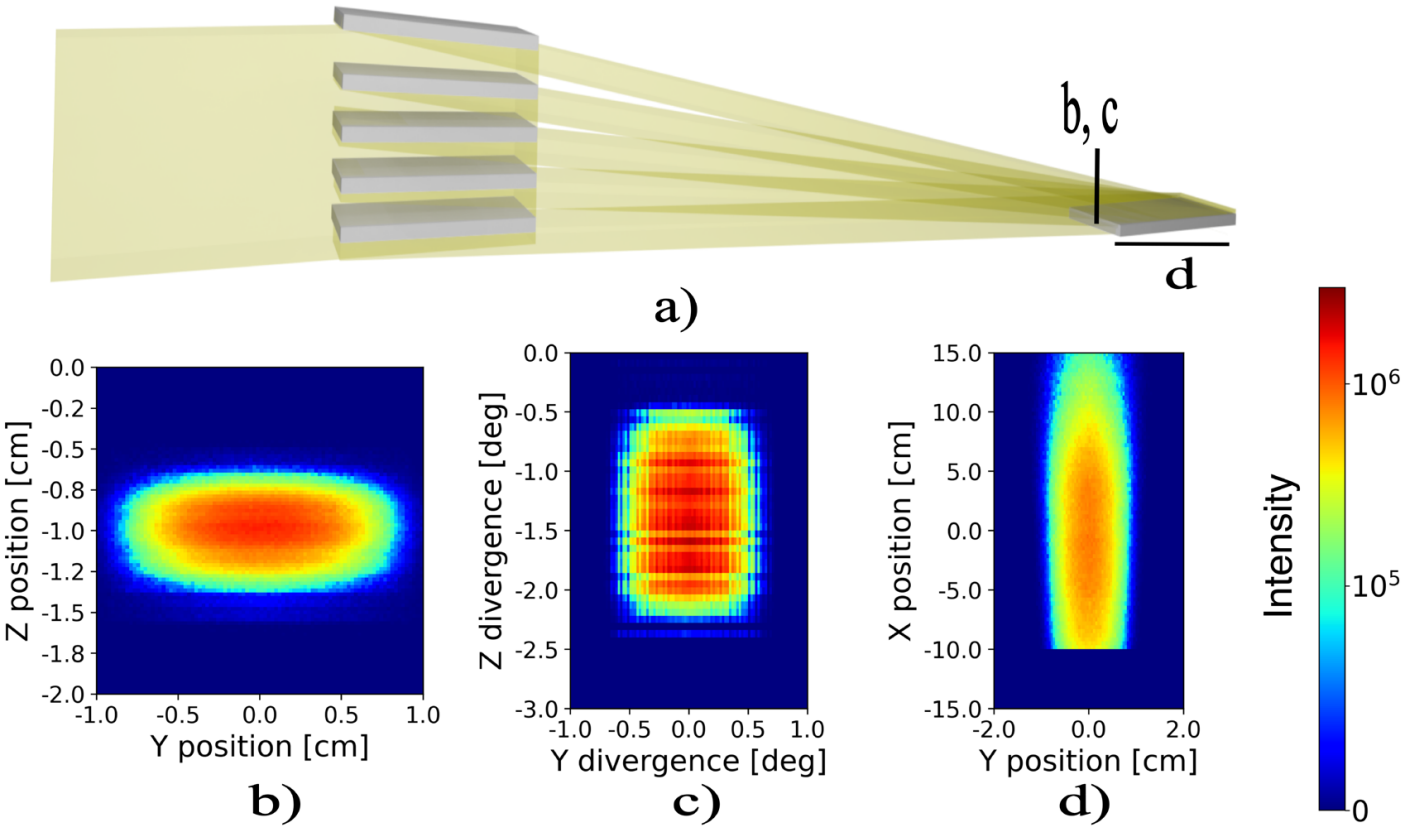
Panel (**a**) displays a sketch of the flat mirror assembly. Panels (**b**) and (**c**) depict the intensity and divergence profiles of the diverted beam, right before the sample. Panel (**d**) Shows the intensity profile along the sample surface.

**Fig. 6:**
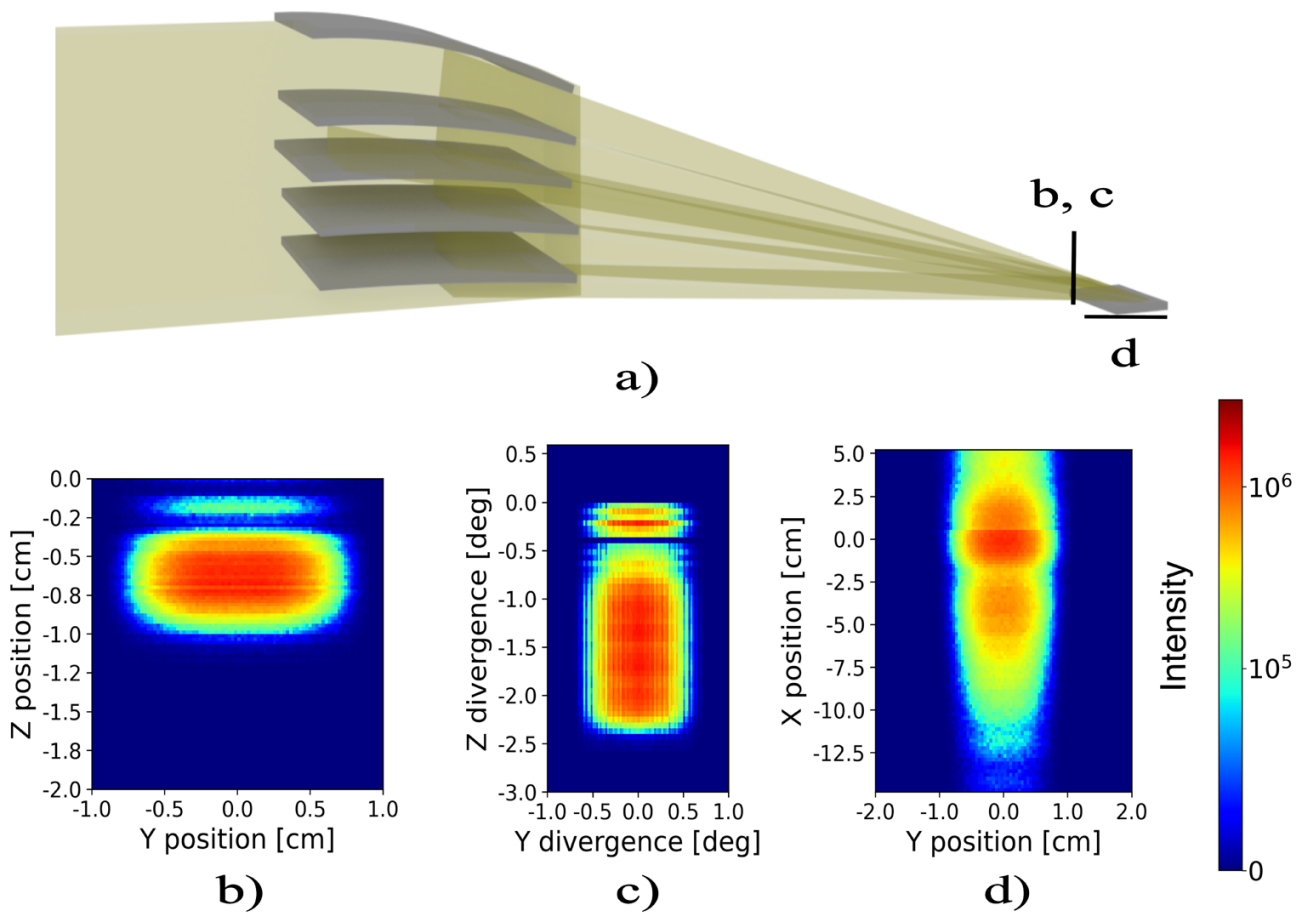
Panel (**a**) displays a sketch of the parabolic mirror assembly. Panels (**b**) and (**c**) depict the intensity and divergence profiles of the diverted beam, right before the sample. Panel (**d**) Shows the intensity profile along the sample surface.

**Fig. 7:**
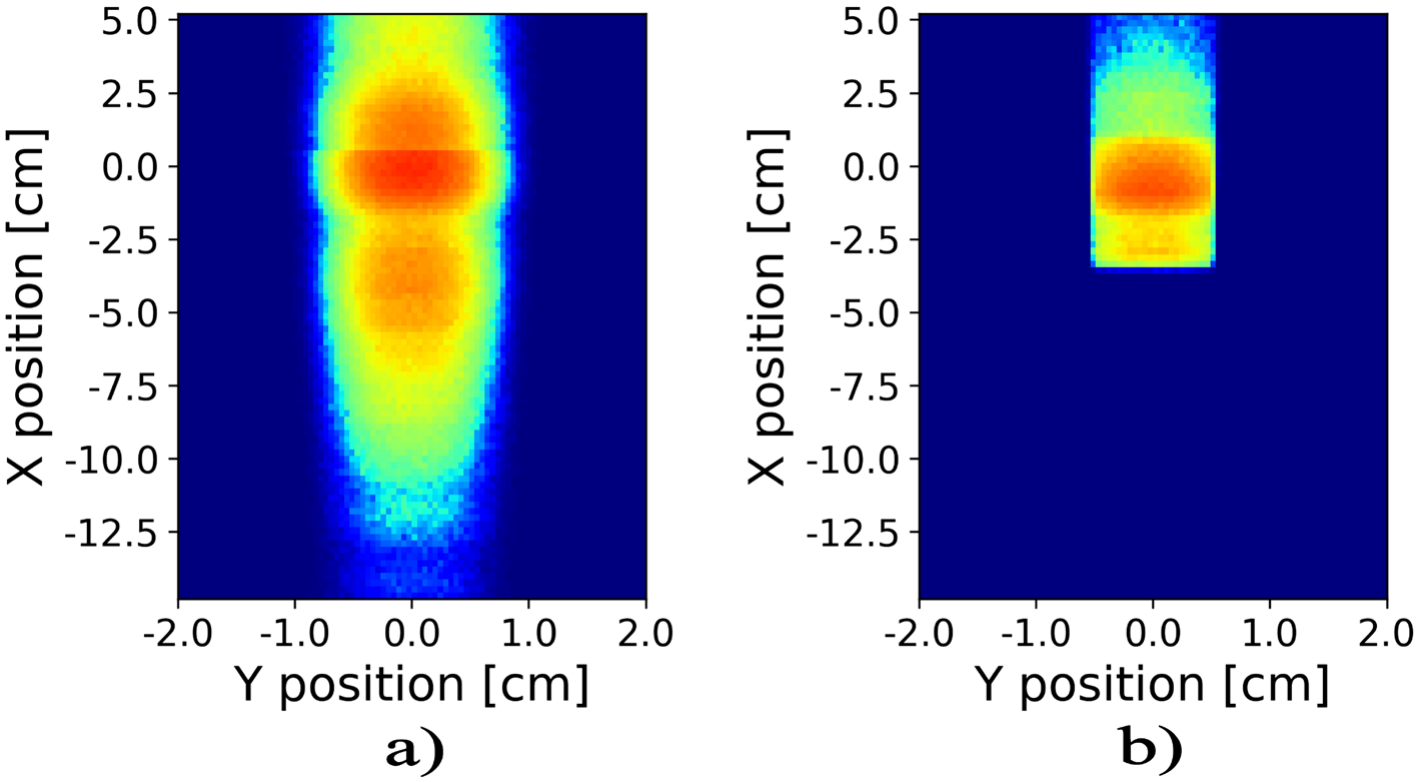
(**a**) Simulated Intensity profile along the sample surface with the parabolic mirror design (**b**) Same simulation as (**a**) but with a slit inserted just before the sample to control the footprint. The slit gap is 1.5 mm in height.

Figures 5 and 6 summarize the results of the ray tracing simulations for the flat and parabolic mirror arrays, respectively. Panels (a) show the mirror stacks and beam path as well as the sample. Panels (b) and (c) show the beam after passing the nested mirror assembly at the position of the sample, as indicated in the sketches (panel a). The width and horizontal divergence of the beam remains unchanged but the height of the beam is reduced, resulting in higher flux. The downwards deflection of the beam is visible as well. For both mirror stacks the vertical divergence is increased from $0.4^\circ$ to $2^\circ$. The features in the divergence around and above 0 degrees for the parabolic mirror stack are an artifact of the simulation, as the parabolic mirror component in Mcstas does not absorb neutrons. Thus transmission and multiple reflections are possible and absorption effects from shadowing of the mirrors are not taken into account. If present in a real device such beam contamination can be removed by slits. Panels (d) show the neutron flux on a sample placed at the focus point with the surface oriented along the horizontal direction. For the parabolic mirror stack the focused beam size along the sample is significantly smaller than for the flat mirror stack confirming better focusing. The flux at the sample is higher for the parabolic stack. It should be noted, however, that there is a significant tail of intensity from out-of-focus neutrons resulting from the incoming divergence in the z-direction (vertical) to the device. Control of the footprint can be done in two ways: One, by a decrease of the incoming vertical divergence, for example, decreasing the vertical divergence to $\pm \ 0.05 ^{\circ }$ (compared to $\pm \ 0.2 ^{\circ }$ in the simulated case) the footprint including out-of-focus tails reduces to 6 cm. Two, slits can be utilized near the sample. In Fig. 7 a slit has been installed just before the sample, excluding most of the out-of-focus neutrons.

**Fig. 8:**
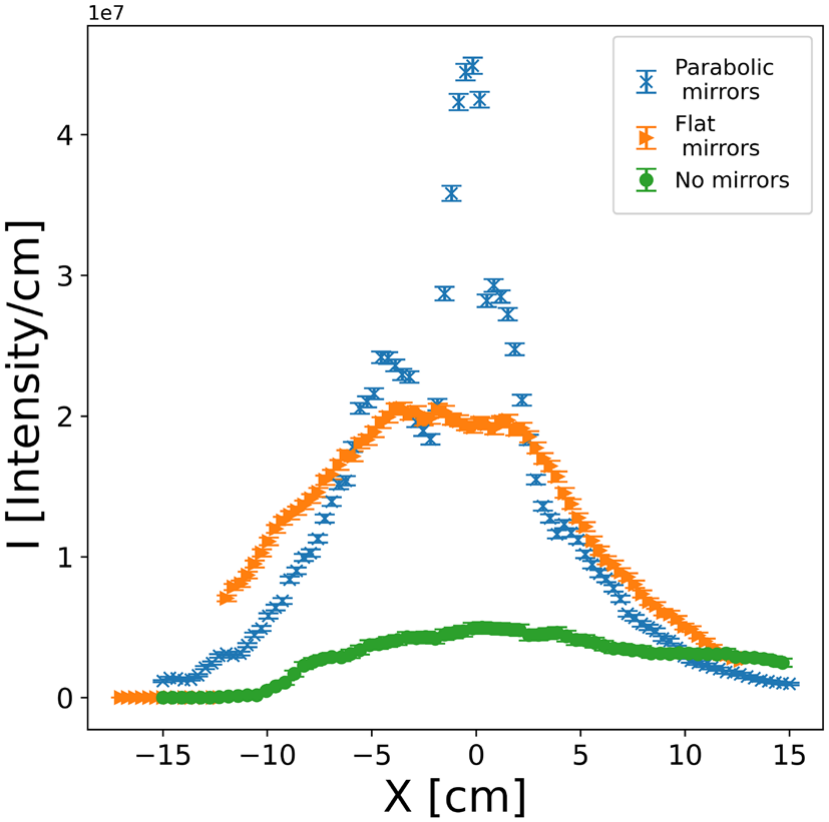
Comparison of intensity profiles (integrated along y direction) at the sample. Blue) Parabolic mirrors, Orange) flat mirrors, green) without mirrors and a tilt of the sample of 0.2 degree.

To get a better idea of the focusing performance of the nested mirror devices Fig. 8 shows the comparison of the beam profiles along a horizontal sample. The intensity is integrated over a sample width of 1 cm along the horizontal direction perpendicular to the direction of the neutron beam. The intensities without any mirrors (green), flat mirror focusing (orange) and parabolic focusing (blue) are plotted. For smaller samples the parabolic mirrors show a significantly better performance. For the case without the mirrors the simulation consisted of tilting the sample by 0.2° relative to the incoming beam (same as used for the mirror simulations), the beam was then reduced in height to 0.05 cm (compared to 1 cm in the case with mirrors), to reduce overillumination of the sample. This setup corresponds to how a standard GISANS measurement could be performed at the Skadi instrument and thus acts as a realistic comparison.

To quantify the gain in neutron flux enabled by the nested mirror optics we have integrated the intensity over a sample area of 5 x 1 cm^2^ and one second assuming a repetition rate of the source of 14 Hz. The average flux at the sample for no, nested flat and nested parabolic mirrors is $1.50*10^{8}$ $n \ cm^{-2} s^{-1}$, $9.43*10^{8}$ $n \ cm^{-2} s^{-1}$ and $1.82*10 ^{9}$ $n \ cm^{-2} s^{-1}$, respectively. The results are summarized in Table 1. By using the focusing optics gain factors at the sample surface between 6 and 12 are achieved for the flat and parabolic nested mirrors, respectively.

**Table 1:** Comparison of neutron flux, simulated for SKADI, hitting a flat 5 cm long sample on a SANS instrument without, with flat and with parabolic nested mirror optics.

	No mirrors	Nested flat mirrors	Nested parabolic mirrors
Flux $n \ cm^{-2} s^{-1}$	$1.50*10^{8}$	$9.43*10^{8}$	$1.82*10^{9}$

**Fig. 9:**
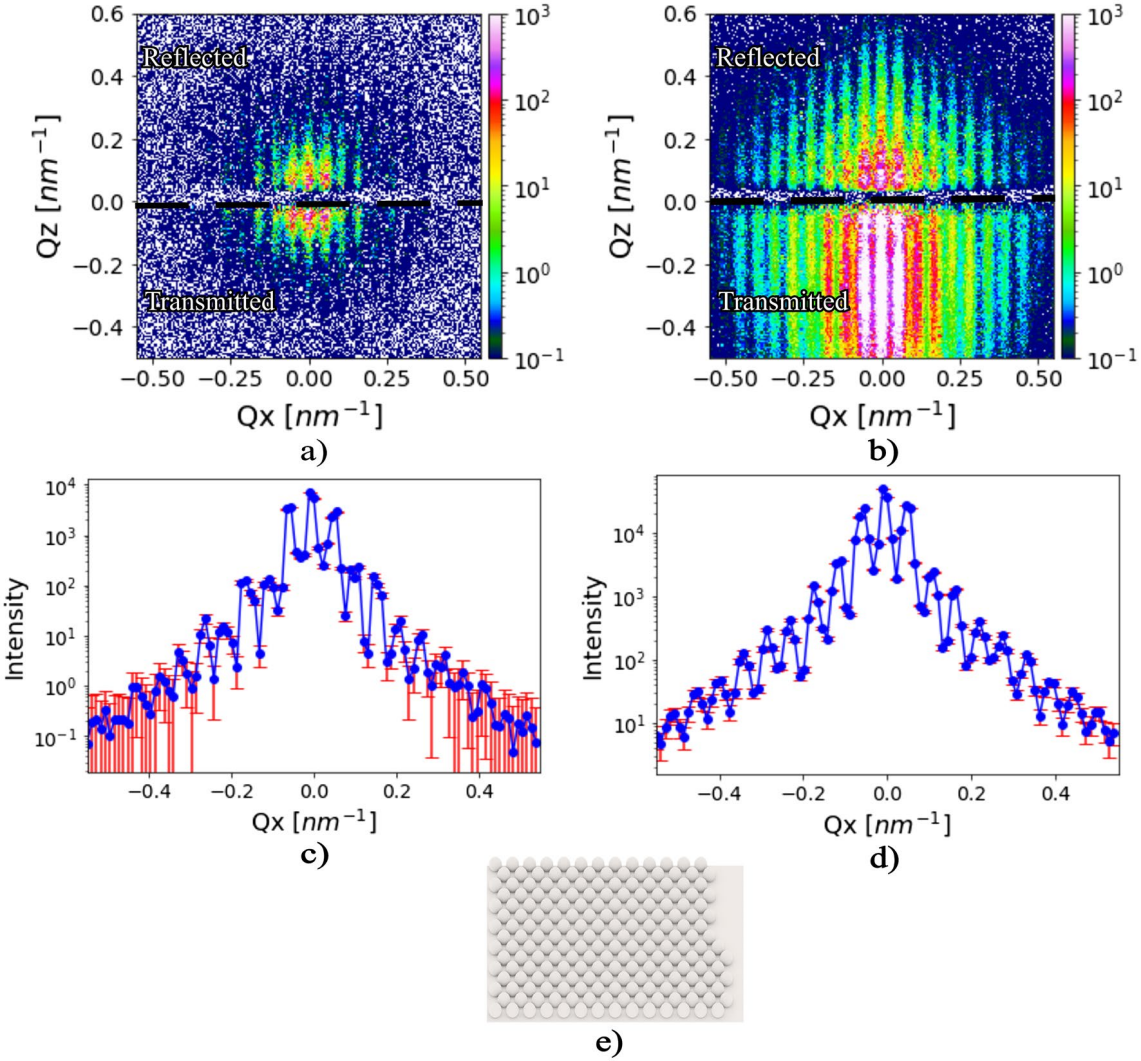
Panels (**a**) and (**b**) show simulated GISANS patterns on the SKADI instrument without and with parabolic nested mirror optics, respectively. The exposure time is 1 second. Panel (**c**) and (**d**) depicts the vertically integrated intensity of panel (**a**) and (**b**). (**e**) Sketch of the simulated sample, showing 74 nm diameter $SiO_2$ nanoparticle assembled into a hexagonal structure with domain size of 300 nm at a $D_2O/Si$ interface.

In order to verify the performance of our nested mirror assembly, we combined ray tracing simulations with grazing incidence scattering simulations in BornAgain^10^ together with a python code package by^40^. A model for a monolayer of hexagonally packed nanoparticles with 74 nm diameter at a $D_2O$/silicon interface was created and simulated using a sample-detector distance of 20 m and a nominal source-to-sample distance of 38.43 m which is comparable to the dimensions of the SKADI instrument. The simulated beam was identical to the one shown earlier but was collimated to a lateral angular divergence of ± $0.05^{\circ }$. Data were simulated for one pulse and upscaled to an acquisition time of 1 second. Figure 9, panel e) represents a sketch of the simulated sample. Panels a) and b) show the scattering from the sample for a conventional GISANS geometry and with the parabolic mirror assembly installed, respectively. The scattered reflected beam (Top half) and scattered transmitted beam (Bottom half) are visible. In between, an empty ”forbidden region” exists because of refraction in the film (for a review of the theory of GISAS see^3,4,41^). Panel c) and d) show the vertically integrated intensities from panels a) and b). The significant improvement of data quality with the parabolic mirrors installed is clearly visible. Note, the excellent data quality for very short aquisition times of just one second. This result suggests that GISANS patterns can be acquired on SKADI very rapidly with the mirror device installed and the study of kinetics will come into reach. It should be noted that the angular range and wavelength bandwidth of the focused beam at the sample results in a range of incoming neutron momenta around the critical value. Scattering of neutrons closer to the critical scattering vector is enhanced due to Yoneda scattering, while neutrons further from the critical value contribute less and potentially contribute equally to the background. Depending on the sample there will be an optimal range for the best signal-to-noise ratio. For liquid interfaces this is important but difficult to estimate in simulations. To tune the angular range there is the possibility of tilting the mirrors, which changes the angular range delivered and moves the focus point, which requires repositioning of the sample. Depending on sample space restrictions this can be used to optimize the measurement for different samples. Also note, the large band width used on SKADI makes the design of our nested mirror optics more challenging than on monochromatic instruments, as operated at reactor sources. On such instruments even larger gains in performance may be expected.

## Conclusion

We have designed a nested mirror device, PortGISANS, focusing a parallel beam onto a horizontal flat sample. The device allows to tune any SANS instrument for GISANS studies of thin films and interfaces. In particular, thin films with light elements, like lipid and block-copolymer films, as well as magnetically structured thin films will gain considerably by our method. Our ray tracing simulations reveal that PortGISANS increases the flux available at a sample by at least a factor of 12 compared to a conventional GISANS geometry. This increase is on the expense of depth resolution. We expect that the decrease in counting time needed to acquire GISANS patterns, will allow time resolved studies of kinetics on time scales down to seconds. The compactness of the device allows the implementation on most SANS instruments, satisfying the space requirements to fit the device in their respective sample area. Another advantage is that the device is easy to align minimizing experimental setup times. In particular the parabolic nested mirror assembly results in a significantly reduced footprint of the beam, allowing smaller sample surfaces. As a next step, we will construct a prototype of PortGISANS with parabolic mirrors and verify its performance under real neutron beam conditions.

## Data Availability

The datasets used and/or analysed during the current study are available from the corresponding author by request. The simulation code for the Mcstas ray-tracing to reproduce the results is freely available at https://github.com/fimehler/Mcstas-Nested-mirror-Optics.git and can be utilized for simulating other instrument configurations.
